# Embedding Undersampling Rotation Forest for Imbalanced Problem

**DOI:** 10.1155/2018/6798042

**Published:** 2018-11-01

**Authors:** Huaping Guo, Xiaoyu Diao, Hongbing Liu

**Affiliations:** School of Computer and Information Technology, Xinyang Normal University, Xinyang 464000, Henan, China

## Abstract

Rotation Forest is an ensemble learning approach achieving better performance comparing to Bagging and Boosting through building accurate and diverse classifiers using rotated feature space. However, like other conventional classifiers, Rotation Forest does not work well on the imbalanced data which are characterized as having much less examples of one class (minority class) than the other (majority class), and the cost of misclassifying minority class examples is often much more expensive than the contrary cases. This paper proposes a novel method called Embedding Undersampling Rotation Forest (EURF) to handle this problem (1) sampling subsets from the majority class and learning a projection matrix from each subset and (2) obtaining training sets by projecting re-undersampling subsets of the original data set to new spaces defined by the matrices and constructing an individual classifier from each training set. For the first method, undersampling is to force the rotation matrix to better capture the features of the minority class without harming the diversity between individual classifiers. With respect to the second method, the undersampling technique aims to improve the performance of individual classifiers on the minority class. The experimental results show that EURF achieves significantly better performance comparing to other state-of-the-art methods.

## 1. Introduction

Imbalanced problem is concerned with the performance of classifiers on the data set with severe class-imbalance distribution, and the problem is often encountered in real world, rising from medical diagnosis, risk management, fraud detection, and other domain applications [1–3]. For two class, the examples are commonly grouped into majority (negative) class or minority (positive) class, and the cost of misclassifying minority class examples is often much more expensive than the negative ones. Take the “mammography” data set as an example, the data set contains 10923 “healthy” patients and 260 “cancerous” patients and how to recognize the “cancerous” patients is very meaningful. However, most conventional classification methods try to achieve high accuracy with the assumption of balanced class distribution, i.e., the number of examples in any class is similar to each other, which leads to the fact that the minority class examples are often ignored and misclassified to the majority class.

Sampling technique is one of the most popular methods to handle the class-imbalance problem, which aims to improve the model performance on class-imbalance data through adjusting data distribution by sampling data space, forcing the model to focus more on the minority class. Examples include undersampling [4, 5], oversampling [6, 7], and SMOTE [8]. Undersampling techniques including random undersampling try to adjust imbalanced distribution by removing the intrinsic examples in the majority class, and on the contrary to undersampling, oversampling techniques learn the model on the rebalanced data by repeating minority class examples. SMOTE including the several improved methods such as borderline-SMOTE [9], safe-level-SMOTE [10], and MWMOTE (Majority Weighted Minority Oversampling TechniquE) [11] is a special version of the oversampling technique, which generates new synthetic examples along the line between the minority examples and their selected nearest neighbors, and the difference of these SMOTE methods lies in how to search the nearest neighbors.

Ensemble learning which has been successfully applied to many applications such as medical detection [12], image recognition [13, 14], and software defect prediction [15] is another effective technique for handling class-imbalanced data [16], where existing efforts roughly fall into the following three categories: (1) bagging-, (2) boosting-, and (3) Rotation Forest-based approaches. Both bagging- and boosting-based approaches often embed sampling techniques into the ensemble learning process. Examples include OverBagging, UnderBagging, UnderOverBagging [17], SMOTEBoost [18, 19], and RUSBoost [20]. The former three methods learn each base classifier on the rebalanced data obtained by sampling the original training data and the last two methods train each base classifier on the adjusted training data distribution obtained by combining the sampling technique with reweighting mechanism of the boosting method. Rotation Forest is a newly proposed ensemble method [21]. The main heuristic is to apply feature extraction and subsequently reconstruct a full feature set for each classifier in the ensemble. This method is also applied to class-imbalance data; for example, Su et al. [22] employed Hellinger distance decision tree (HDDT) [23, 24] instead of C4.5 or CART as the base learner of Rotation Forest to deal with class-imbalance issues. Hosseinzadeh and Eftekharia [25] preprocessed the original data using the fuzzy cluster and synthetic oversampling technique (SMOTE) to obtain the training set on which Rotation Forest is learned. Fang et al. [26] learned and preprocessed the training set before learning each rotation matrix. All of these Rotation Forest methods train individual classifiers on the whole training set.

This paper proposes a simple but effective Embedding Undersampling Rotation Forest (EURF) method to handle class-imbalance problem. The main heuristics differing from Rotation Forest consists of (1) undersampling subsets from majority class for learning projection matrices, (2) re-undersampling majority class to construct balanced training sets, and (3) learning classifiers from the training sets in the spaces defined by the corresponding matrices. For the first step, the undersampling technique is mainly to obtaining diverse rotation matrices, with the goal of learning individual classifiers with diversity. With respect to the second step, the undersampling method aims to balance the training distribution, improving the performance of each individual classifier on the minority class. Following Rotation Forest, the decision tree learning method is used to train the base classifiers because it is sensitive to the rotation of feature axes. Experimental results show that when compared with other state-of-the-art methods, EURF shows significantly better performance on measures of recall, *g*-mean, *f*-measure, and AUC.

The rest of this paper is grouped as follows: after presenting ensemble methods for the imbalanced problem in Section 2, Section 3 describes the Embedding Undersampling Rotation Forest method for the problem, followed by presenting the experimental results in Section 4, and finally, Section 5 concludes this work.

## 2. Ensemble for Imbalanced Problem

An ensemble refers to a group of base learners whose decisions are aggregated with the goal of achieving better performance than their constituent members [16, 27]. Ensemble methods are also successfully applied to the imbalanced problem, where existing efforts can mainly be categorized into three groups: (1) bagging-, (2) boosting-, and (3) Rotation Forest-based methods.

### 2.1. Bagging-Based Approaches

Bagging [28, 29] is a parallel-based ensemble, which trains an individual classifier on a bootstrap sample of the original data set and produces the output by combining the votes of individual classifiers using majority voting.  Algorithm 1 shows the pseudocode for bagging.

Many approaches have been proposed using bagging to handle the imbalanced problem due to its simplicity and good generalization performance [30]. The class-imbalance-oriented bagging differs from conventional bagging mainly in how to manipulate data to generate new training sets for training individual classifiers (line 3 in Algorithm 1). For example, OverBagging [17] oversamples the original data set instead of random sampling the set into a bag for learning the individual classifier to overcome the class imbalance. As a special version of OverBagging, SMOTEBagging [16] oversamples minority class examples by generating synthetic examples of the minority class. On the contrary to OverBagging, UnderBagging [31] employs undersampling techniques instead of oversampling ones to create diverse bags to learn individual classifiers. The UnderBagging method has been used with different names, but maintaining the same functional structure, such as asymmetric bagging [32] and roughly-balanced bagging [33]. UnderOverBagging [17] employs both oversampling and undersampling to create diverse bags; a resampling rate is set in each iteration, which determines the example number from each class. DTE_SBD [34] adopts differentiated sampling rates, respectively, for the positive class and the negative class with different principles. SMOTE is used for increasing the samples of the minority positive class without repeating, and bagging is used for drawing the majority negative subset with certain degrees of diversity.

As a special version of UnderBagging, EasyEnsemble [4, 35] undersamples several subsets from majority class, trains an individual classifier on each subset, and combines the outputs of individual classifiers. Unlike UnderBagging, the base classifier of EasyEnsemble is learned by the ensemble learning method Adaboost instead of the single model. Therefore, EasyEnsemble is an ensemble of ensembles, and AdaBoost is mainly to reduce bias while bagging mainly reduces variance.

### 2.2. Boosting-Based Approaches

Boosting introduced by Freund and Schapire [36] is a family of methods, and AdaBoost is the most prominent member. AdaBoost sequentially trains each individual classifier. After each iteration, AdaBoost gives more bias (weights) on the examples that are hard to be correctly classified by the classifiers, forcing subsequent classifiers to focus more on them.  Algorithm 2 shows the pseudocode for Adaboost.

Boosting-based ensembles [30] have been also introduced to handle the class-imbalance problem by embedding data preprocessing into boosting learning procedure. These methods try to alter and bias distribution such that the following classifiers focus more on the minority class every iteration. For example, SMOTEBoost [18, 19] introduces synthetic examples of the minority class using the SMOTE data preprocessing algorithms. The weights of the new examples are proportional to the total number of examples in the new data set. RUSBoost [20] performs similarly to SMOTEBoost with exception of removing examples from the majority class in each iteration. Adaptive EUSBoost [37] learns a Real Adaboost on each undersampled data. Besides, Adaptive EUSBoost embeds cost-sensitive weight modification and adaptive boundary decision strategy into the learning process for improving the model performance on class-imbalance data.

Like AdaBoost, BalanceCascade [4, 35] trains individual classifiers sequentially, and for each iteration, it removes the majority class examples that are correctly classified with high confidence, namely, the examples are not taken into account in further iterations. Like EasyEnsemble, BalanceCascade trains each individual classifier using AdaBoost, and therefore, BalanceCascade is a hybrid model, namely, an ensemble of ensembles.

### 2.3. Rotation Forest-Based Approaches

Rotation Forest [21] is an effective ensemble method, more robust comparing to bagging, boosting, and Random Forest [38] by creating individual classifiers with high accuracy and diversity. The main heuristic is to apply feature extraction to subsets of features and reconstruct a full feature set for each classifier in the ensemble: randomly splitting the feature set into *K* disjoint subsets, randomly selecting a nonempty subset of classes, running principal component analysis (PCA) on each subset with a bootstrap sample of training set, and then organizing all the principal components in a sparse rotation matrix **R**. A classifier is trained on the whole training set in the feature space defined by **R**. Decision tree C4.5 [39] is selected as the base learner as it is sensitive to feature rotation.  Algorithm 3 shows the pseudocode for Rotation Forest.

Rotation Forest has been also applied to the imbalanced problems, for example, Su et al. [22] used Hellinger distance decision tree (HDDT) [23, 24] instead of C4.5 to train individual classifiers on whole training set. Hosseinzadeh and Eftekharia [25] learned Rotation Forest on the data obtained by preprocessing training set using the synthetic oversampling technique (SMOTE) [8] and fuzzy cluster [40]. Fang et al. [26] learned the rotation matrices on data sets obtained by randomly undersampling or oversampling (SMOTE) the training set, and each base classifier is constructed on the whole training set.

This paper proposes an improved Rotation Forest based on the undersampling technique for the class-imbalance problem. Unlike conventional Rotation Forest-based approaches, the proposed method learns both rotation matrices and individual classifiers on diverse balanced data sets obtained by undersampling the original data instead of on the whole data set or on the same data.

## 3. Improving Rotation Forest via Undersampling Technique

### 3.1. Algorithm

**Figure 1:**
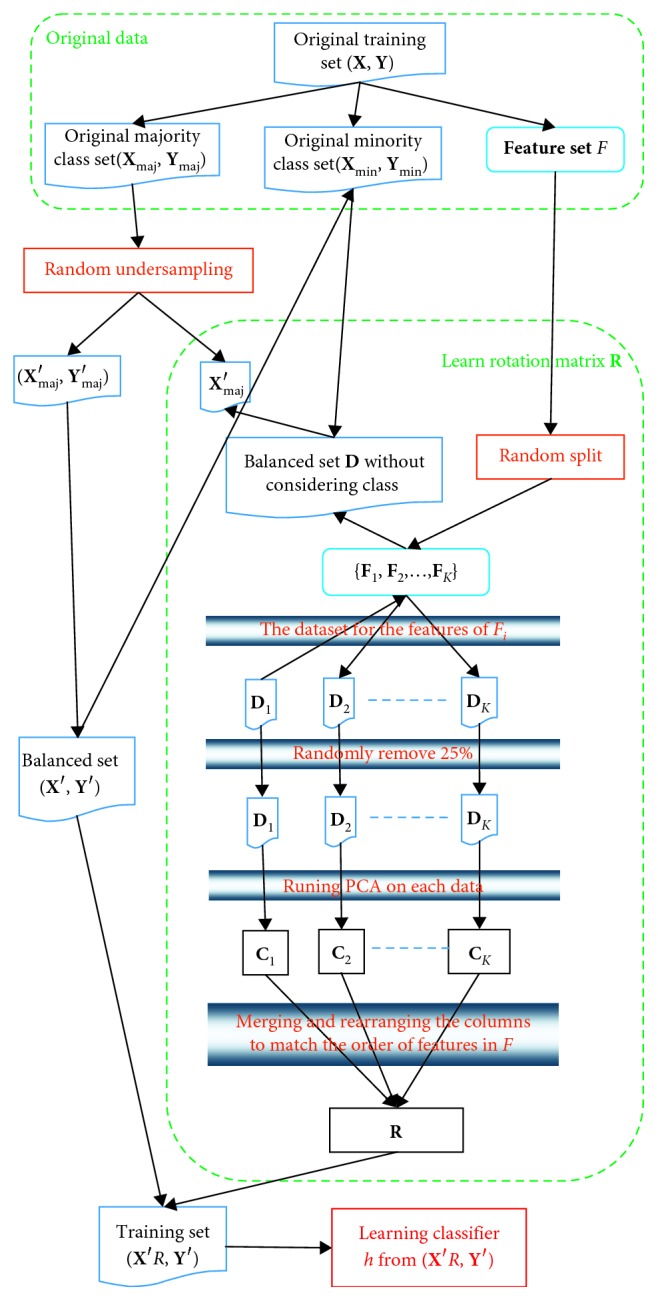
The process of learning a classifier of the proposed method.

Class-imbalance problem often exists in many applications, and this problem causes that conventional classifier learning methods do not work well [1–3]. This section proposes a novel Rotation Forest called Embedding Undersampling Rotation Forest (EURF) to handle class-imbalance problem. EURF differs from the conventional ensemble method for learning each individual classifier in the following two aspects: (1) undersampling a balanced subset with the size of any class equal to each other for learning a rotation matrix and (2) obtaining a training set by projecting a re-undersampled balanced subset into the feature space defined by the rotation matrix and learning an individual classifier on the training set. The process of learning each individual classifier *h* is shown in Figure 1.

Let **x**=[*x*_1_, *x*_2_,…, *x*_*n*_] be a data point described by *n* features and let **X** be the data set containing objects in a form of an *N* × *n* matrix. Suppose **Y**=[*y*_1_, *y*_2_,…, *y*_*n*_] is a vector of class labels for the data and *y*_*i*_ is either majority class or minority class, i.e., *y*_*i*_ ∈ {maj, min}. As shown in Figure 1, EURF first splits the original training set (**X**, **Y**) into two disjoint sets based on the class labels, namely, majority class set (**X**_maj_, **Y**_maj_) and minority class set (**X**_maj_, **Y**_maj_). Then, EURF mainly learns the rotation matrix **R** using the following four steps:

- Constructing the balanced data **D** by merging the minority set **X**_min_ and the undersampled subset **X**_maj_′ of majority class set, |**X**_min_|=|**X**_maj_′|
- Randomly splitting feature set **F** into disjoint subsets {**F**_*j*_|*j*=1,2,…*K*}
- Removing 25% of each data subset **D**_*i*_ of **D** corresponding to the feature subset **F**_*i*_ and running PCA on **D**_*i*_ to obtain subrotation matrix **C**_*i*_
- Constructing rotation matrix **R** by merging and rearranging the columns of each **C**_*i*_ to match the order of features in **F**

After the learning rotation matrix **R**, EURF learns a classifier *h* on balanced data (**X**′, **Y**′) in the rotation feature space defined by **R**, namely, learning *h* from (**X**′**R**,**Y**′), where (**X**′, **Y**′) is obtained by merging minority class set (**X**_min_, **Y**_min_) and undersampled subset (**X**_maj_′, **Y**_maj_′) of the majority class set and |**X**_min_|=|**X**_maj_′|.

Algorithm 4 shows the pseudocode for EURF. The differences with Rotation Forest (refers to Algorithm 3) are mainly shown in lines 4∼5 and lines 14∼15. Lines 4 and 5 are to sample a subset **X**_maj,*i*_ from majority class set **X**_maj_ (without considering class labels) to obtain balanced set, |**X**_maj,*i*_|=|**X**_min_|. Thus, the rotation matrix **R**_*i*_ are learned on a balanced data set. Lines 14∼15 aim to reconstruct the balanced data using the same method as lines 4∼5 with exception of considering class label. Therefore, the base classifier *h*_*i*_ is also learned from a balanced data set. Besides, unlike conventional Rotation Forest which selects and eliminates a random nonempty subspace of classes (line 6 in Algorithm 3), EURF does not handle classes due to the limit data set for learning the rotation matrix.

In this paper, decision tree is selected as the base learner due to its sensitiveness to the rotation of the feature axes. PCA [41] is also used for learning the rotation matrix following Rotation Forest [21].

### 3.2. Discussion

Dietterich stated [42] “A necessary and sufficient condition for an ensemble of classifiers to be more accurate than any of its individual members is if the classifiers are accurate and diverse.” For class-imbalance problem, similar issues should be addressed: (1) the high *accuracy* of each individual classifier on minority class and (2) the high *diversity* between individual classifiers.

- *Accuracy*. The accuracy of EURF (the proposed method) on the minority class is guaranteed by the undersampling technique through the following two approaches: (1) undersampling the majority class to construct balanced data sets on which individual classifiers are constructed, forcing the learned classifiers to focus more on the minority class (lines 14∼16, Algorithm 4) and (2) re-undersample the majority class to construct balanced data sets for training rotation matrices, and thus the matrices capture more information from the minority class, enhancing the accuracy of individual classifiers on the minority class (lines 7∼11, Algorithm 4). In this way, EURF learns individual classifiers with high accuracy on the minority class.
- *Diversity*. Diversity is a key to the success of an ensemble, and the diversity of EURF mainly comes from the learning process of both rotation matrices and individual classifier. Two approaches of learning rotation matrices make sure the ensemble diversity: (1) the splitting method of feature subsets (line 6, Algorithm 1) and (2) the sampling technique used for manipulating the distribution of data set (lines 4, 5, and 9, Algorithm 1). For the first approach, according to Reference [21], the number of different partitions of the feature set into *K* subsets of size *M* is *T* = *n*!/[*K*!(*M*!)^*k*^]. If the size of the ensemble is *L*, the probability that all classifiers will be different is *T*!/[(*T*−*L*)!*T*^*L*^]. For the ensemble with 50 member, if *n* = 9 and *K* = 3, the probability that all classifiers are different from each other is less than 0.01. Therefore, an extra randomization of the ensemble is meaningful. In this study, PCA is applied to a bootstrap sample of **X**_*i*_ to increase the diversity of ensemble members. For the second method, the sampling technique is used to create balanced data for training rotation matrix. Therefore, the larger the ratio between the size of the majority class and that of the minority class set is, the larger the diversity between individual classifiers is. Besides, for the learning process of individual classifiers, the sampling technique is reused to create balanced data to the training base classifier, which further improves the ensemble diversity (lines 14∼16, Algorithm 4).

## 4. Experiments

### 4.1. Evaluation Measure

Evaluation measure is extremely essential to assess the effectiveness of an algorithm, and for imbalanced problem, precision, recall, *f*-measure, *g*-mean, and AUC are the most frequently used ones. The examples classified by a classifier can be grouped into four categories as shown in Table 1, and thus the precision and recall are defined as

(1) $$\begin{array}{c} \text{precision}=\frac{\text{TP}}{\text{TP}+\text{TF}},\\ \text{recall}=\frac{\text{TP}}{\text{TP}+\text{FN}}. \end{array}$$

*f* − measure is a harmonic mean between recall and precision. Specifically, *f* − measure is defined as

(2) $$\begin{array}{c} f-\text{measure}=\frac{\left(1+\delta^{2}\right)\times \text{recall}\times \text{precision}}{\delta^{2}\times \text{recall}+\text{precision}}, \end{array}$$

where *δ*, often set to be 1 (*f*1 − measure), is a coefficient to adjust the relative importance of precision versus recall.

Like *f*-measure, *g* − mean is another metric considering both normal class and abnormal class. Specifically, *g* − mean measures the balanced performance of a classifier using the geometric mean of the recall of the abnormal class and that of the normal class. Formally, *g* − mean is as follows:

(3) $$\begin{array}{c} g-\text{mean}=\sqrt{\frac{\text{TA}}{\text{TA}+\text{FN}}\times \frac{\text{TN}}{\text{TN}+\text{FA}}}. \end{array}$$

Besides, AUC is a commonly used measure to evaluate models' performances. According to [43], AUC can be estimated by

(4) $$\begin{array}{c} \text{AUC}=\frac{\left(\left(\text{TP}/\left(\text{TP}+\text{FN}\right)\right)+\left(\text{TN}/\left(\text{TN}+\text{FP}\right)\right)\right)}{2}. \end{array}$$

In this paper, recall, *f* − measure, *g* − mean, and AUC are employed to evaluate the classification performance on imbalanced data sets.

### 4.2. Experimental Setup

Thirty-one imbalanced data sets were selected from the KEEL lab [44], and the details about the sets are shown Table 2, where #Attrs, #Exps, and #IR are the attribute number, example number, and imbalance ratio defined as the ratio between the size of the majority class set and that of the majority class set. All the data sets are two-class imbalanced ones, and the imbalanced degree of the sets varies from 41.4 (highly imbalanced) to 3.2 (only slightly imbalanced). In this section, 5 × 2-fold cross-validation strategy was conducted to evaluate the performance of the proposed method EURF [45, 46]. We perform five replications of a two-fold cross-validation. In each replication, the available data are partitioned into two random equal-sized sets. Each learning algorithm is trained on one set at a time and tested on the other set.

Ten methods were selected as candidates to test the performance of the proposed method EURF:

- Undersampling-C45 (UC) [47] preprocessed training sets using the random undersampling technique to obtain relatively balanced data set and then learned a model using C4.5 [39] on the balanced set.
- Oversampling-C45 (OC) [47]. On contrary to UC, OC used the random oversampling technique to obtain relatively balanced data set.
- UnderBagging (UBG) [31] learned each member on the undersampled subset from the majority class. C4.5 was selected as the base learner. The number of members *T* was set to be 40.
- OverBagging (OBG) [17] learned each member on the rebalanced data by randomly oversampling training set, and C4.5 was selected as the base classifier learner. The number of member *T* was set to be 40.
- RUSBoost (UBT) [20] combined the undersample technique into the boost learning process. The number of member *T* was set to be 40, and for each member, a subset with size equal to the minority class was sampled (without replacement) from the majority class. Then, C4.5 was used to train a classifier using the subset and minority class set.
- EasyEnsemble (EE) [4, 35] samples *T* subsets from the majority class and trains an Adaboost with *J* weak learner using each of them. We selected C4.5 as weak classifiers and set *T* = 4 and *J* = 10.
- BalanceCascade (BC) [4, 35] is similar to EasyEnsemble exception of removing correctly classified major class examples from further consideration. C4.5 was selected to train weak classifiers, and we set *T* = 4 and *J* = 10.
- *DTE_SBD* [34] constructs decision tree ensemble based on SMOTE, undersampling and differentiated sampling rates (DSR). The number of individual classifiers was set to be 40. The *k*-nearest neighbor parameter of SMOTE was set to 5, and C4.5 was selected to train weak classifiers.
- Rotation Forest [21] constructs decision tree ensemble based on feature projection and sampling technique (Algorithm 3). C4.5 [39] is selected as the base learner, and we set *T* = 40.
- *EURF* is the proposed method in this paper. Here, we set *T* = 40, namely, the number of bases classifier is 40. C4.5 was used to train base classifiers (Algorithm 4).

All of the above methods were implemented in our data mining tool “LySpoon” and please contact hpguo@xynu.edu.cn for the related source code.

### 4.3. Experimental Results

Tables 3–6, respectively, report the summary results of the comparing methods on the measures of recall, *g*-mean, *f*1-measure, and AUC, where the results in the parentheses are the algorithm ranks defined as follows [45, 48]: on a data set, the best performing algorithm gets the rank of 1.0, the second best rank 2.0, and so on. In case of ties, average ranks are assigned. The results with bold type of these tables are the corresponding algorithm (row) with the best performance on the corresponding data (column).

Table 3 shows that EURF performs the best AUC on 23 out of the 29 data sets and outperforms (is outperformed by) DTE_SBD, BC, EE, UBT, UBG, OBg, UC, OC, and RF on 26(3), 29(0), 27(2), 29(0), 26(3), 28(1), 28(1), 29(0), 28(1), 27(2), and 27(2) out of the data sets. Unlike Tables 3 and 4 show that EURF performs similar to BC, EE, and UBG, and significantly better than other methods, which indicates that EURF tries to improve the model recall while keeping the high accuracy of the model.  Table 5 reports the summary results and the ranks of the nine algorithms on the measure of *g*-mean. Similar to the results on AUC and recall, Table 5 shows that EURF performs best *g*-mean on 23 out of the 29 data sets and outperforms (is outperformed by) DTE_SBD, BC, EE, UBT, UBG, OBg, UC, and OC on 27(2), 26(3), 27(2), 29(0), 26(3), 27(2), 29(0), 28(1), and 26(3) out of the data sets.  Table 6 shows the experimental results of compared algorithms on measure of *f*1-measure. From Table 6, EURF performs similar to RF and significantly outperforms other methods. Combining with the results of Table 4, EURF improves the recall of RF at the expense of RF's accuracy.

Table 7 shows the average performance including average rank in parentheses of each method on measures of AUC, recall, *g*-mean, and *f*1-measure. From Table 7, EURF outperforms other methods on the comprehensive measures including AUC, *g*-mean, and *f*1-measure. Table 7 also shows that EURF performs similar to BC, EE, and UBG and outperforms other methods on recall.

The Friedman test, a nonparametric statistical test, was used to ensure whether the superiority of our methods is by accident. The Friedman test can be used to detect differences across multiple algorithms based on the ranks of algorithms on multiple data sets. STAC [49], a web platform for the comparison of algorithms using statistical tests, was used for the experiments. We assume that the performances of all of the nine methods for comparison are the same and set the *p* value at 0.05. The experimental result shows that the hypothesis of all of the algorithms with the same performance on measures of AUC, recall, *g*-mean, and *f*1-measure is rejected, with an extremely low *p* value (*p* < 0.00001).

**Figure 2:**
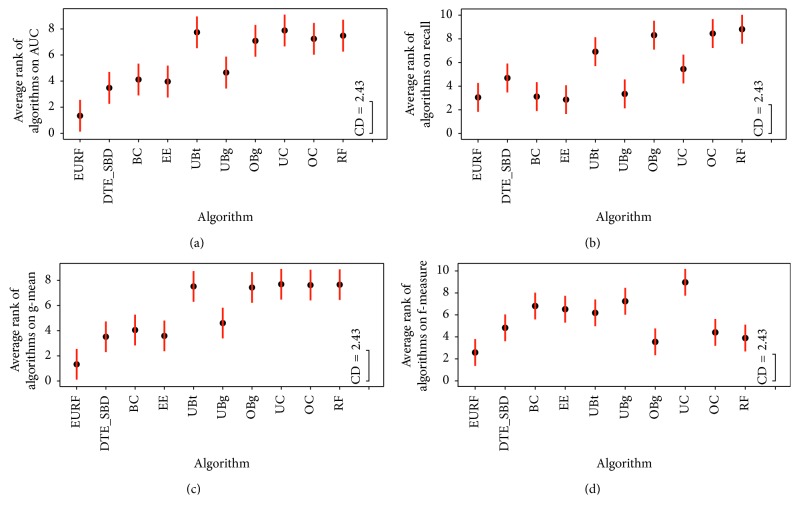
The Nemenyi figures of comparing algorithms on (a) AUC, (b) recall, (c) *g*-mean, and (d) *f*1-measure.

To further differentiate these algorithms, the Nemenyi post hoc test was also adopted after the hypothesis “the performance of the comparisons on the groups of data is similar” is rejected. Nemenyi computes an average ranking difference threshold CD, and the hypothesis “the performance of two algorithms is the same” will be rejected if their average ranking difference is larger than CD/2 [19], where CD is defined as

(5) $$\begin{array}{c} \text{CD}=q_{\alpha }\sqrt{\frac{k\left(k+1\right)}{6N}}, \end{array}$$

where *k* is the number of algorithms, *N* is the number of data sets, and *q*_*α*_ is the critical range of the Tukey distribution. We set *α*=0.05.  Figure 2 shows the Nemenyi figure of comparing algorithms on the measure of AUC, recall, *g*-mean, and *f*-measure. From Figures 2(a), 2(c), and 2(d), EURF significantly outperforms all of the other nine methods on measures of AUC, *g*-mean, and *f*1-measure. From Figure 2(b), EURF performs similar to BC, EE, and UBG and significantly outperforms all of the other nine methods.

## 5. Conclusion

In this paper, we proposed a novel Rotation Forest method called Embedding Undersampling Rotation Forest (EURF) based on undersampling technique with the difference in both learning the rotation matrices and individual classifiers: (1) undersampling subsets from the majority class for constructing balanced training sets on which the rotate matrices are built and (2) learning individual classifiers on balanced data obtained by projecting re-undersampling subsets of the original training set to new spaces. Therefore, EURF mainly obtains the diversity between ensemble members through feature projection and undersampling. Experimental results show that EURF significantly outperforms other state-of-the-art methods for imbalanced data sets on the measure of recall, *g*-mean, *f*-measure, and AUC.

## Figures and Tables

**Algorithm 1:**
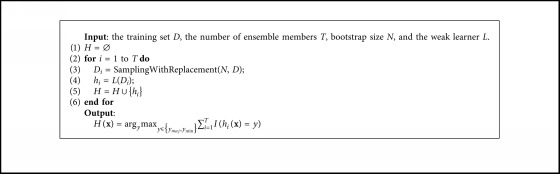
Bagging.

**Algorithm 2:**
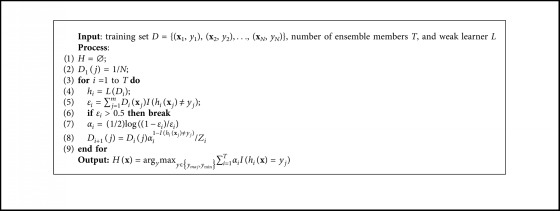
AdaBoost.

**Algorithm 3:**
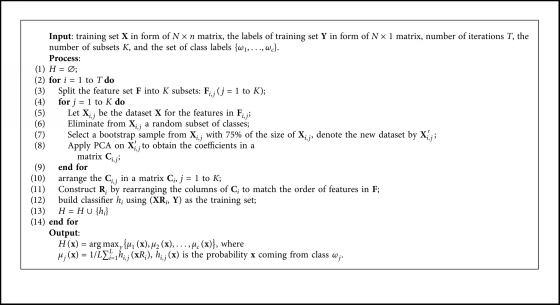
Rotation Forest.

**Algorithm 4:**
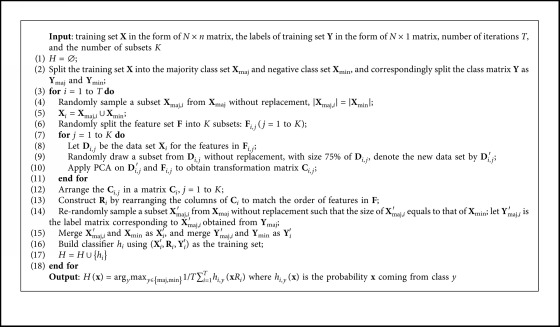
Embedding Undersampling Rotation Forest for imbalanced problem.

**Table 1:** Confusion matrix.

	Predicted as positive	Predicted as negative
Actually positive	TP	FP
Actually negative	FN	TN

**Table 2:** The data sets used in this paper.

ID	Name	#Attr	#Exps	#IR
id1	glass-0-1-2-3_vs_4-5-6	9	214	3.2
id2	vehicle0	18	846	3.25
id3	ecoli1	7	336	3.36
id4	new-thyroid1	5	215	5.14
id5	new-thyroid2	5	215	5.14
id6	ecoli2	7	336	5.46
id7	segment0	19	2308	6.02
id8	glass6	9	214	6.38
id9	ecoli3	7	336	8.6
id10	page-blocks0	10	5472	8.79
id11	yeast-2_vs_4	8	514	9.08
id12	yeast-0-5-6-7-9_vs_4	8	528	9.35
id13	vowel0	13	988	9.98
id14	glass-0-1-6_vs_2	9	192	10.29
id15	glass2	9	214	11.59
id16	yeast-1_vs_7	7	459	14.3
id17	glass4	9	214	15.47
id18	ecoli4	7	336	15.8
id19	abalone9-18	8	731	16.4
id20	dermatology-6	34	358	16.9
id21	shuttle-c2-vs-c4	9	129	20.5
id22	yeast-1-4-5-8_vs_7	8	693	22.1
id23	yeast-2_vs_8	8	482	23.1
id24	flare-F	11	1066	23.79
id25	car-good	6	1728	24.04
id26	yeast4	8	1484	28.1
id27	yeast5	8	1484	32.73
id28	ecoli-0-1-3-7_vs_2-6	7	281	39.14
id29	yeast6	8	1484	41.4

**Table 3:** The AUC and ranks of comparing methods.

ID	EURF	DTE_SBD	BC	EE	UBT	UBG	OBG	UC	OC	RF
id1	**0.9296**(1.0)	0.9051(5.0)	0.9130(3.0)	0.9117(4.0)	0.9013(6.0)	0.8993(7.0)	0.8771(9.0)	0.8801(8.0)	0.8718(10.0)	0.9256(2.0)
id2	**0.9682**(1.0)	0.9410(6.0)	0.9562(3.0)	0.9555(4.0)	0.9332(7.0)	0.9428(5.0)	0.9251(8.0)	0.9176(10.0)	0.9180(9.0)	0.9640(2.0)
id3	**0.8895**(1.0)	0.8812(3.0)	0.8647(5.0)	0.8589(6.0)	0.8506(7.0)	0.8861(2.0)	0.8401(9.0)	0.8753(4.0)	0.8486(8.0)	0.8234(10.0)
id4	**0.9694**(1.0)	0.9316(5.0)	0.9478(3.0)	0.9508(2.0)	0.9238(8.0)	0.9147(9.0)	0.9307(6.0)	0.9108(10.0)	0.9251(7.0)	0.9440(4.0)
id5	**0.9667**(1.0)	0.9503(5.0)	0.9606(3.0)	0.9620(2.0)	0.9416(7.0)	0.9440(6.0)	0.9237(9.0)	0.9247(8.0)	0.9187(10.0)	0.9526(4.0)
id6	**0.9057**(1.0)	0.8824(5.0)	0.8870(2.0)	0.8852(3.0)	0.8718(6.0)	0.8842(4.0)	0.8606(7.0)	0.8452(10.0)	0.8522(9.0)	0.8596(8.0)
id7	**0.9916**(1.0)	0.9840(8.0)	0.9907(2.0)	0.9906(3.0)	0.9880(5.0)	0.9821(9.0)	0.9870(6.0)	0.9797(10.0)	0.9855(7.0)	0.9891(4.0)
id8	**0.9356**(1.0)	0.9224(2.0)	0.9077(5.0)	0.9085(4.0)	0.9062(6.0)	0.9027(7.0)	0.8949(9.0)	0.8668(10.0)	0.9090(3.0)	0.9005(8.0)
id9	**0.8899**(1.0)	0.8682(3.0)	0.8402(5.0)	0.8545(4.0)	0.7696(8.0)	0.8761(2.0)	0.7766(7.0)	0.8081(6.0)	0.7598(9.0)	0.7439(10.0)
id10	**0.9610**(1.0)	0.9565(4.0)	0.9594(2.0)	0.9583(3.0)	0.9444(6.0)	0.9543(5.0)	0.9356(7.5)	0.9356(7.5)	0.9261(9.0)	0.9257(10.0)
id11	0.9272(3.0)	**0.9338**(1.0)	0.9254(4.0)	0.9313(2.0)	0.8578(9.0)	0.9222(5.0)	0.8703(7.0)	0.9086(6.0)	0.8681(8.0)	0.8467(10.0)
id12	**0.8059**(1.0)	0.7991(2.0)	0.7802(5.0)	0.7895(4.0)	0.6149(10.0)	0.7978(3.0)	0.7062(8.0)	0.7696(6.0)	0.7102(7.0)	0.6620(9.0)
id13	0.9775(2.0)	0.9616(6.0)	0.9673(5.0)	0.9681(4.0)	0.9742(3.0)	0.9530(8.0)	0.9613(7.0)	0.9318(10.0)	0.9529(9.0)	**0.9827**(1.0)
id14	**0.7170**(1.0)	0.6273(2.0)	0.5750(6.0)	0.5561(8.0)	0.5742(7.0)	0.6233(3.0)	0.6207(4.0)	0.5431(9.0)	0.6015(5.0)	0.4983(10.0)
id15	**0.7162**(1.0)	0.6668(2.0)	0.6538(3.0)	0.5951(7.0)	0.5492(8.0)	0.6386(4.0)	0.6181(5.0)	0.5484(9.0)	0.6017(6.0)	0.5000(10.0)
id16	**0.7532**(1.0)	0.7236(2.0)	0.7047(3.0)	0.6964(5.0)	0.5780(9.0)	0.7013(4.0)	0.6075(7.0)	0.6378(6.0)	0.6028(8.0)	0.5495(10.0)
id17	**0.8504**(1.0)	0.8229(3.0)	0.7606(7.0)	0.8251(2.0)	0.7057(10.0)	0.7920(4.0)	0.7856(5.5)	0.7071(9.0)	0.7856(5.5)	0.7205(8.0)
id18	**0.9209**(1.0)	0.8991(3.0)	0.8946(4.0)	0.9022(2.0)	0.8644(7.0)	0.8751(5.0)	0.8408(8.0)	0.7900(10.0)	0.8330(9.0)	0.8681(6.0)
id19	**0.7851**(1.0)	0.7223(3.0)	0.6952(5.0)	0.6971(4.0)	0.5153(10.0)	0.7326(2.0)	0.6133(9.0)	0.6292(7.0)	0.6371(6.0)	0.6146(8.0)
id20	**0.9979**(1.0)	0.9852(2.0)	0.9787(4.5)	0.9772(6.0)	0.9723(10.0)	0.9787(4.5)	0.9762(7.5)	0.9725(9.0)	0.9762(7.5)	0.9826(3.0)
id21	0.9976(4.0)	0.8167(7.0)	0.8492(5.0)	0.8433(6.0)	0.8000(8.5)	0.8000(8.5)	**1.0000**(2.0)	0.7667(10.0)	**1.0000**(2.0)	**1.0000**(2.0)
id22	0.6386(2.0)	0.6255(5.0)	0.6373(3.0)	**0.6446**(1.0)	0.4600(10.0)	0.6309(4.0)	0.5431(8.0)	0.5903(6.0)	0.5520(7.0)	0.5030(9.0)
id23	**0.7544**(1.0)	0.7500(3.0)	0.7309(5.0)	0.7303(6.0)	0.7014(8.0)	0.7219(7.0)	0.7457(4.0)	0.6739(9.0)	0.7514(2.0)	0.6598(10.0)
id24	0.8265(3.0)	**0.8399**(1.0)	0.8088(5.0)	0.8208(4.0)	0.4965(10.0)	0.8268(2.0)	0.6663(7.0)	0.7869(6.0)	0.6613(8.0)	0.4995(9.0)
id25	**0.9625**(1.0)	0.9110(5.0)	0.9381(3.0)	0.9411(2.0)	0.9005(6.0)	0.9304(4.0)	0.8518(7.0)	0.8402(9.0)	0.8517(8.0)	0.6600(10.0)
id26	**0.8443**(1.0)	0.8379(2.0)	0.8259(5.0)	0.8271(4.0)	0.5924(9.0)	0.8290(3.0)	0.6686(8.0)	0.8064(6.0)	0.6699(7.0)	0.5424(10.0)
id27	0.9588(2.0)	**0.9592**(1.0)	0.9518(5.0)	0.9535(4.0)	0.8987(7.0)	0.9560(3.0)	0.8650(9.0)	0.9413(6.0)	0.8669(8.0)	0.7952(10.0)
id28	**0.8553**(1.0)	0.7989(3.0)	0.7725(4.0)	0.7693(5.0)	0.6838(9.0)	0.8123(2.0)	0.6916(8.0)	0.7460(6.0)	0.6923(7.0)	0.5667(10.0)
id29	**0.8781**(1.0)	0.8751(2.0)	0.8493(5.0)	0.8521(4.0)	0.7746(8.0)	0.8750(3.0)	0.7821(7.0)	0.8334(6.0)	0.7682(9.0)	0.6886(10.0)

**Table 4:** The recalls and ranks of comparing methods.

ID	EURF	DTE_SBD	BC	EE	UBT	UBG	OBg	UC	OC	RF
id1	**0.9218**(1.0)	0.8863(6.0)	0.9180(2.0)	0.9178(3.0)	0.8665(7.0)	0.8943(4.0)	0.8192(9.0)	0.8472(8.0)	0.8038(10.0)	0.8906(5.0)
id2	**0.9930**(1.0)	0.9487(5.0)	0.9739(3.0)	0.9769(2.0)	0.9236(8.0)	0.9628(4.0)	0.9016(9.0)	0.9308(7.0)	0.8936(10.0)	0.9437(6.0)
id3	0.9041(3.0)	0.9092(2.0)	0.8729(5.0)	0.8598(6.0)	0.7745(8.0)	**0.9327**(1.0)	0.7744(9.0)	0.8881(4.0)	0.8022(7.0)	0.7009(10.0)
id4	0.9556(3.0)	0.8987(6.0)	**0.9657**(1.0)	0.9650(2.0)	0.8765(8.0)	0.8984(7.0)	0.8758(9.0)	0.9039(4.0)	0.8647(10.0)	0.8990(5.0)
id5	0.9546(3.0)	0.9373(5.0)	**0.9601**(1.0)	0.9595(2.0)	0.9154(7.0)	0.9490(4.0)	0.8585(9.5)	0.9261(6.0)	0.8585(9.5)	0.9141(8.0)
id6	0.8769(4.0)	0.8500(6.0)	0.8846(2.5)	**0.8923**(1.0)	0.8000(7.0)	0.8846(2.5)	0.7769(8.0)	0.8538(5.0)	0.7615(9.0)	0.7346(10.0)
id7	0.9860(3.0)	0.9806(8.0)	**0.9897**(1.5)	**0.9897**(1.5)	0.9818(6.5)	0.9818(6.5)	0.9824(5.0)	0.9830(4.0)	0.9781(10.0)	0.9787(9.0)
id8	**0.9100**(1.0)	0.8967(3.0)	0.9019(2.0)	0.8819(4.0)	0.8610(7.5)	0.8681(6.0)	0.8395(9.0)	0.8610(7.5)	0.8743(5.0)	0.8205(10.0)
id9	0.9039(2.0)	0.8699(4.0)	0.8464(6.0)	0.8752(3.0)	0.6402(7.0)	**0.9157**(1.0)	0.6131(8.0)	0.8474(5.0)	0.5833(9.0)	0.5144(10.0)
id10	**0.9775**(1.0)	0.9603(5.0)	0.9721(2.0)	0.9714(3.0)	0.9131(7.0)	0.9667(4.0)	0.8902(8.0)	0.9367(6.0)	0.8755(9.0)	0.8616(10.0)
id11	0.9252(5.0)	0.9492(2.0)	0.9411(3.0)	**0.9529**(1.0)	0.7420(9.0)	0.9260(4.0)	0.7729(7.0)	0.8942(6.0)	0.7689(8.0)	0.7068(10.0)
id12	0.7686(4.0)	0.7731(3.0)	0.7642(5.0)	**0.7958**(1.0)	0.5526(7.0)	0.7922(2.0)	0.4782(9.0)	0.7451(6.0)	0.4942(8.0)	0.3445(10.0)
id13	**1.0000**(1.0)	0.9689(4.0)	0.9844(2.0)	0.9800(3.0)	0.9556(7.0)	0.9622(6.0)	0.9311(9.0)	0.9467(8.0)	0.9156(10.0)	0.9667(5.0)
id14	0.6319(3.0)	0.5542(7.0)	0.5819(5.0)	0.5694(6.0)	0.6222(4.0)	0.6375(2.0)	0.3181(8.0)	**0.7153**(1.0)	0.2819(9.0)	0.0000(10.0)
id15	0.6708(2.0)	0.5986(6.0)	**0.7069**(1.0)	0.6292(4.0)	0.6000(5.0)	0.6458(3.0)	0.3042(8.0)	0.4847(7.0)	0.2694(9.0)	0.0000(10.0)
id16	**0.7333**(1.0)	0.6867(5.0)	0.6933(4.0)	0.7000(2.5)	0.5333(7.0)	0.7000(2.5)	0.2733(9.0)	0.6400(6.0)	0.2867(8.0)	0.1000(10.0)
id17	**0.8500**(1.0)	0.8000(3.5)	0.7095(5.5)	0.8333(2.0)	0.6905(7.0)	0.8000(3.5)	0.6000(8.5)	0.7095(5.5)	0.6000(8.5)	0.4500(10.0)
id18	0.8900(4.0)	0.9000(3.0)	**0.9100**(1.5)	0.9100(1.5)	0.7800(7.0)	0.8800(5.0)	0.7000(9.0)	0.8300(6.0)	0.6900(10.0)	0.7400(8.0)
id19	**0.7286**(1.0)	0.6429(5.0)	0.6619(4.0)	0.6714(3.0)	0.5857(6.0)	0.7095(2.0)	0.2667(9.0)	0.5429(7.0)	0.3238(8.0)	0.2333(10.0)
id20	**1.0000**(1.0)	0.9900(3.5)	0.9900(3.5)	0.9900(3.5)	0.9600(9.0)	0.9900(3.5)	0.9600(9.0)	0.9800(6.0)	0.9600(9.0)	0.9700(7.0)
id21	**1.0000**(2.5)	0.6333(7.0)	0.7000(6.0)	0.7333(5.0)	0.6000(8.5)	0.6000(8.5)	**1.0000**(2.5)	0.5333(10.0)	**1.0000**(2.5)	**1.0000**(2.5)
id22	0.6133(6.0)	0.5933(7.0)	0.7067(2.0)	**0.7200**(1.0)	0.6667(5.0)	0.6733(4.0)	0.1200(9.0)	0.6867(3.0)	0.1667(8.0)	0.0067(10.0)
id23	0.6200(5.0)	0.6200(5.0)	0.6600(2.0)	**0.7000**(1.0)	0.5500(7.0)	0.6200(5.0)	0.5200(9.0)	0.6400(3.0)	0.5400(8.0)	0.3200(10.0)
id24	0.8154(6.0)	0.8379(4.0)	0.8342(5.0)	0.8385(3.0)	0.5952(7.0)	**0.8701**(1.0)	0.4331(8.0)	0.8654(2.0)	0.4242(9.0)	0.0000(10.0)
id25	0.9795(5.0)	0.9441(6.0)	0.9943(2.0)	0.9942(3.0)	0.9885(4.0)	**1.0000**(1.0)	0.7401(9.0)	0.9249(7.0)	0.7403(8.0)	0.3223(10.0)
id26	0.8460(6.0)	0.8535(4.0)	0.8580(3.0)	0.8462(5.0)	0.7195(7.0)	**0.8735**(1.0)	0.3645(9.0)	0.8615(2.0)	0.3715(8.0)	0.0862(10.0)
id27	0.9773(6.0)	0.9864(2.5)	0.9818(4.5)	0.9864(2.5)	0.8227(7.0)	**1.0000**(1.0)	0.7409(9.0)	0.9818(4.5)	0.7455(8.0)	0.5955(10.0)
id28	0.8083(2.0)	0.7417(5.5)	0.7750(3.5)	0.7750(3.5)	0.6500(7.0)	**0.8333**(1.0)	0.4000(8.5)	0.7417(5.5)	0.4000(8.5)	0.1333(10.0)
id29	0.8572(5.0)	0.8690(3.0)	0.8801(2.0)	0.8683(4.0)	0.7879(7.0)	**0.8863**(1.0)	0.5837(8.0)	0.8510(6.0)	0.5605(9.0)	0.3801(10.0)

**Table 5:** The *g*-means and ranks of comparing methods.

ID	EURF	DTE_SBD	BC	EE	UBT	UBG	OBg	UC	OC	RF
id1	**0.9223**(1.0)	0.9040(5.0)	0.9126(3.0)	0.9110(4.0)	0.8997(6.0)	0.8981(7.0)	0.8735(9.0)	0.8784(8.0)	0.8672(10.0)	0.9217(2.0)
id2	**0.9679**(1.0)	0.9408(6.0)	0.9560(3.0)	0.9552(4.0)	0.9330(7.0)	0.9424(5.0)	0.9247(8.0)	0.9172(10.0)	0.9173(9.0)	0.9637(2.0)
id3	**0.8883**(1.0)	0.8793(3.0)	0.8633(5.0)	0.8572(6.0)	0.8437(8.0)	0.8838(2.0)	0.8356(9.0)	0.8728(4.0)	0.8453(7.0)	0.8120(10.0)
id4	**0.9688**(1.0)	0.9289(5.0)	0.9469(3.0)	0.9497(2.0)	0.9195(7.5)	0.9120(9.0)	0.9266(6.0)	0.9077(10.0)	0.9195(7.5)	0.9418(4.0)
id5	**0.9662**(1.0)	0.9497(5.0)	0.9604(3.0)	0.9617(2.0)	0.9397(7.0)	0.9434(6.0)	0.9193(9.0)	0.9239(8.0)	0.9147(10.0)	0.9513(4.0)
id6	**0.9048**(1.0)	0.8812(5.0)	0.8862(2.0)	0.8848(3.0)	0.8666(6.0)	0.8838(4.0)	0.8550(7.0)	0.8436(10.0)	0.8450(9.0)	0.8495(8.0)
id7	**0.9916**(1.0)	0.9840(8.0)	0.9907(2.0)	0.9906(3.0)	0.9879(5.0)	0.9821(9.0)	0.9870(6.0)	0.9796(10.0)	0.9854(7.0)	0.9890(4.0)
id8	**0.9351**(1.0)	0.9218(2.0)	0.9065(5.0)	0.9077(3.0)	0.9047(6.0)	0.9016(7.0)	0.8908(9.0)	0.8656(10.0)	0.9071(4.0)	0.8963(8.0)
id9	**0.8886**(1.0)	0.8664(3.0)	0.8375(5.0)	0.8518(4.0)	0.7459(8.0)	0.8727(2.0)	0.7540(7.0)	0.8014(6.0)	0.7260(9.0)	0.7037(10.0)
id10	**0.9609**(1.0)	0.9565(4.0)	0.9593(2.0)	0.9582(3.0)	0.9438(6.0)	0.9542(5.0)	0.9344(8.0)	0.9355(7.0)	0.9246(9.0)	0.9233(10.0)
id11	0.9266(3.0)	**0.9330**(1.0)	0.9249(4.0)	0.9305(2.0)	0.8487(9.0)	0.9214(5.0)	0.8631(7.0)	0.9065(6.0)	0.8611(8.0)	0.8343(10.0)
id12	**0.8041**(1.0)	0.7976(2.0)	0.7780(5.0)	0.7873(4.0)	0.5291(10.0)	0.7967(3.0)	0.6664(8.0)	0.7665(6.0)	0.6743(7.0)	0.5747(9.0)
id13	0.9772(2.0)	0.9616(6.0)	0.9670(5.0)	0.9680(4.0)	0.9739(3.0)	0.9529(8.0)	0.9607(7.0)	0.9313(10.0)	0.9519(9.0)	0.9823(1.0)
id14	**0.7009**(1.0)	0.6121(3.0)	0.5652(4.0)	0.5369(5.0)	0.5061(7.0)	0.6147(2.0)	0.5210(6.0)	0.3847(9.0)	0.4940(8.0)	0.0000(10.0)
id15	**0.7042**(1.0)	0.6544(2.0)	0.6276(3.0)	0.5727(5.0)	0.4607(9.0)	0.6273(4.0)	0.5243(7.0)	0.5349(6.0)	0.4909(8.0)	0.0000(10.0)
id16	**0.7495**(1.0)	0.7211(2.0)	0.7004(3.0)	0.6930(5.0)	0.5371(7.0)	0.6977(4.0)	0.5016(8.0)	0.6277(6.0)	0.4969(9.0)	0.2612(10.0)
id17	**0.8310**(1.0)	0.8023(3.0)	0.7399(7.0)	0.8048(2.0)	0.6562(9.0)	0.7722(4.0)	0.7438(5.5)	0.6720(8.0)	0.7438(5.5)	0.6440(10.0)
id18	**0.9179**(1.0)	0.8977(3.0)	0.8925(4.0)	0.8996(2.0)	0.8439(7.0)	0.8739(5.0)	0.8230(8.0)	0.7730(10.0)	0.8134(9.0)	0.8525(6.0)
id19	**0.7779**(1.0)	0.7142(3.0)	0.6897(5.0)	0.6946(4.0)	0.4639(10.0)	0.7307(2.0)	0.4919(8.0)	0.6127(6.0)	0.5447(7.0)	0.4744(9.0)
id20	**0.9979**(1.0)	0.9850(2.0)	0.9784(4.5)	0.9769(6.0)	0.9718(10.0)	0.9784(4.5)	0.9755(7.5)	0.9722(9.0)	0.9755(7.5)	0.9823(3.0)
id21	0.9975(4.0)	0.6577(7.0)	0.6992(6.0)	0.7310(5.0)	0.6394(8.5)	0.6394(8.5)	**1.0000**(2.0)	0.5971(10.0)	**1.0000**(2.0)	**1.0000**(2.0)
id22	0.6323(2.0)	0.6196(5.0)	0.6236(3.0)	0.6329(1.0)	0.3361(8.0)	0.6220(4.0)	0.3179(9.0)	0.5401(6.0)	0.3894(7.0)	0.0257(10.0)
id23	**0.7308**(1.0)	0.7255(2.0)	0.7109(4.0)	0.7131(3.0)	0.6585(8.0)	0.6981(6.0)	0.6975(7.0)	0.6554(9.0)	0.7091(5.0)	0.5160(10.0)
id24	0.8239(2.5)	0.8389(1.0)	0.8051(5.0)	0.8181(4.0)	0.4022(9.0)	0.8239(2.5)	0.6129(7.0)	0.7778(6.0)	0.6046(8.0)	0.0000(10.0)
id25	**0.9619**(1.0)	0.9050(5.0)	0.9364(3.0)	0.9395(2.0)	0.8954(6.0)	0.9278(4.0)	0.8399(7.5)	0.8330(9.0)	0.8399(7.5)	0.5210(10.0)
id26	**0.8416**(1.0)	0.8342(2.0)	0.8236(5.0)	0.8249(3.0)	0.5459(9.0)	0.8241(4.0)	0.5910(8.0)	0.7998(6.0)	0.5946(7.0)	0.2563(10.0)
id27	0.9581(2.0)	**0.9587**(1.0)	0.9512(5.0)	0.9528(4.0)	0.8894(7.0)	0.9550(3.0)	0.8511(9.0)	0.9399(6.0)	0.8532(8.0)	0.7655(10.0)
id28	**0.8393**(1.0)	0.7734(3.0)	0.7401(4.0)	0.7341(5.0)	0.6438(7.0)	0.7852(2.0)	0.5527(9.0)	0.7000(6.0)	0.5533(8.0)	0.1633(10.0)
id29	**0.8766**(1.0)	0.8740(3.0)	0.8468(5.0)	0.8504(4.0)	0.7416(8.0)	0.8742(2.0)	0.7538(7.0)	0.8309(6.0)	0.7382(9.0)	0.5989(10.0)

**Table 6:** The *f*1-measures and ranks of comparing methods.

ID	EURF	DTE_SBD	BC	EE	UBT	UBG	OBg	UC	OC	RF
id1	0.8515(2.0)	0.8330(4.0)	0.8308(5.0)	0.8284(6.0)	0.8387(3.0)	0.8136(7.0)	0.8064(8.0)	0.7964(10.0)	0.8034(9.0)	**0.8835**(1.0)
id2	0.9125(2.0)	0.8762(5.0)	0.8961(3.0)	0.8918(4.0)	0.8755(6.0)	0.8699(8.0)	0.8718(7.0)	0.8309(10.0)	0.8591(9.0)	**0.9463**(1.0)
id3	**0.7797**(1.0)	0.7579(4.0)	0.7410(8.0)	0.7360(10.0)	0.7645(2.0)	0.7552(5.0)	0.7390(9.0)	0.7585(3.0)	0.7435(7.0)	0.7441(6.0)
id4	**0.9368**(1.0)	0.8639(5.0)	0.8401(8.0)	0.8565(7.0)	0.8631(6.0)	0.8034(9.0)	0.8947(3.0)	0.7808(10.0)	0.8858(4.0)	0.9178(2.0)
id5	0.9259(2.0)	0.8887(6.0)	0.8933(5.0)	0.8968(3.0)	0.8816(7.0)	0.8462(9.0)	0.8934(4.0)	0.8016(10.0)	0.8712(8.0)	**0.9322**(1.0)
id6	0.7882(2.0)	0.7395(5.0)	0.7173(7.0)	0.6994(9.0)	0.7591(3.0)	0.7073(8.0)	0.7486(4.0)	0.6309(10.0)	0.7343(6.0)	**0.8069**(1.0)
id7	0.9845(2.0)	0.9541(8.0)	0.9707(4.0)	0.9704(5.0)	0.9735(3.0)	0.9411(9.0)	0.9669(7.0)	0.9255(10.0)	0.9679(6.0)	**0.9877**(1.0)
id8	0.8441(2.0)	0.8075(3.0)	0.7429(9.0)	0.7707(7.0)	0.7944(4.0)	0.7675(8.0)	0.7811(6.0)	0.6559(10.0)	0.7871(5.0)	**0.8442**(1.0)
id9	**0.6133**(1.0)	0.5807(3.0)	0.5242(8.0)	0.5413(6.0)	0.5178(9.0)	0.5581(5.0)	0.5730(4.0)	0.4445(10.0)	0.5363(7.0)	0.5860(2.0)
id10	0.7936(7.0)	0.8086(5.0)	0.7970(6.0)	0.7926(8.0)	0.8588(3.0)	0.7807(9.0)	0.8654(2.0)	0.7468(10.0)	0.8416(4.0)	**0.8827**(1.0)
id11	0.7216(5.0)	0.7107(6.0)	0.6837(10.0)	0.6919(9.0)	0.7496(2.0)	0.7019(7.0)	0.7477(3.0)	0.6978(8.0)	0.7438(4.0)	**0.7722**(1.0)
id12	**0.4753**(1.0)	0.4557(3.0)	0.4201(7.0)	0.4176(8.0)	0.2862(10.0)	0.4385(6.0)	0.4594(2.0)	0.4167(9.0)	0.4547(4.0)	0.4437(5.0)
id13	0.8179(5.0)	0.7997(7.0)	0.7967(8.0)	0.8119(6.0)	0.9431(2.0)	0.7638(9.0)	0.9244(3.0)	0.6872(10.0)	0.9094(4.0)	**0.9762**(1.0)
id14	**0.3459**(1.0)	0.2415(4.0)	0.1932(7.0)	0.1802(8.0)	0.2019(6.0)	0.2239(5.0)	0.3033(2.0)	0.1786(9.0)	0.2678(3.0)	0.0000(10.0)
id15	**0.3086**(1.0)	0.2573(4.0)	0.2244(5.0)	0.1869(7.0)	0.1593(9.0)	0.2148(6.0)	0.2995(2.0)	0.1670(8.0)	0.2724(3.0)	0.0000(10.0)
id16	**0.3016**(1.0)	0.2781(2.0)	0.2452(5.0)	0.2353(6.0)	0.2081(8.0)	0.2472(4.0)	0.2584(3.0)	0.1998(9.0)	0.2351(7.0)	0.1707(10.0)
id17	0.4155(4.0)	0.3961(5.0)	0.3475(7.0)	0.3591(6.0)	0.2655(10.0)	0.3050(8.0)	**0.5817**(1.5)	0.2690(9.0)	**0.5817**(1.5)	0.5364(3.0)
id18	0.6918(3.0)	0.5315(7.0)	0.4987(8.0)	0.5332(6.0)	0.6084(5.0)	0.4687(9.0)	0.6973(2.0)	0.3325(10.0)	0.6583(4.0)	**0.8127**(1.0)
id19	0.3441(2.0)	0.2634(5.0)	0.2163(8.0)	0.2175(7.0)	0.1188(10.0)	0.2496(6.0)	0.2710(4.0)	0.1892(9.0)	0.3022(3.0)	**0.3504**(1.0)
id20	**0.9679**(1.0)	0.8739(6.0)	0.7962(7.5)	0.7836(9.0)	0.8796(5.0)	0.7962(7.5)	0.9212(3.5)	0.7773(10.0)	0.9212(3.5)	0.9475(2.0)
id21	0.9571(4.0)	0.6500(7.0)	0.6857(5.0)	0.6534(6.0)	0.6300(8.5)	0.6300(8.5)	**1.0000**(2.0)	0.5800(10.0)	**1.0000**(2.0)	**1.0000**(2.0)
id22	**0.1376**(1.0)	0.1314(2.0)	0.1284(5.0)	0.1303(3.0)	0.0777(9.0)	0.1263(7.0)	0.1265(6.0)	0.1123(8.0)	0.1300(4.0)	0.0111(10.0)
id23	0.3449(5.0)	0.3199(6.0)	0.2467(8.0)	0.2184(9.0)	0.3744(4.0)	0.2518(7.0)	0.4690(1.0)	0.2119(10.0)	0.4433(2.0)	0.4421(3.0)
id24	0.2889(2.0)	**0.3001**(1.0)	0.2416(5.0)	0.2586(3.0)	0.1090(9.0)	0.2556(4.0)	0.2229(6.0)	0.2070(8.0)	0.2149(7.0)	0.0000(10.0)
id25	**0.5962**(1.0)	0.3847(7.0)	0.4164(6.0)	0.4286(5.0)	0.3159(9.0)	0.3751(8.0)	0.5608(2.0)	0.2486(10.0)	0.5591(3.0)	0.4330(4.0)
id26	0.2712(3.0)	0.2546(4.0)	0.2262(6.0)	0.2369(5.0)	0.1168(10.0)	0.2246(7.0)	**0.3402**(1.0)	0.2040(8.0)	0.3269(2.0)	0.1467(9.0)
id27	0.4996(5.0)	0.4740(6.0)	0.4417(8.0)	0.4420(7.0)	0.6356(4.0)	0.4155(9.0)	**0.7028**(1.0)	0.3928(10.0)	0.6964(2.0)	0.6696(3.0)
id28	0.3351(4.0)	0.3317(5.0)	0.2558(9.0)	0.2672(6.0)	0.2634(7.0)	0.2562(8.0)	0.3793(2.0)	0.3376(3.0)	**0.3960**(1.0)	0.1600(10.0)
id29	0.2869(4.0)	0.2589(5.0)	0.1917(10.0)	0.2033(8.0)	0.2458(6.0)	0.2388(7.0)	0.4879(2.0)	0.1961(9.0)	0.4453(3.0)	**0.4889**(1.0)

**Table 7:** The average performance (including rank) of comparing methods on measures of AUC, recall, *g*-mean, and *f*1-measure.

Measure	EURF	DTE_SBD	BC	EE	UBT	UBG	OBg	UC	OC	RF
AUC	0.8819 (1.34)	0.8544 (3.48)	0.8457 (4.12)	0.8468 (3.97)	0.7774 (7.74)	0.8477 (4.66)	0.8057 (7.09)	0.8058 (7.88)	0.8034 (7.24)	0.7644 (7.48)
Recall	0.8655 (3.05)	0.8304 (4.69)	0.8489 (3.12)	0.8546 (2.86)	0.7571 (6.91)	0.8501 (3.34)	0.6496 (8.31)	0.8122 (5.45)	0.6495 (8.45)	0.5384 (8.81)
*g*-mean	0.8774 (1.33)	0.8441 (3.52)	0.8352 (4.05)	0.8376 (3.59)	0.7424 (7.52)	0.8376 (4.60)	0.7651 (7.43)	0.7856 (7.69)	0.7649 (7.62)	0.6346 (7.66)
*f*1-measure	0.6048 (2.59)	0.5525 (4.83)	0.5245 (6.81)	0.5255 (6.52)	0.5281 (6.19)	0.5182 (7.24)	0.6170 (3.55)	0.4751 (8.97)	0.6065 (4.41)	0.5825 (3.90)

## Data Availability

The data sets used in this paper can be obtained from the KEEL lab (http://sci2s.ugr.es/keel/download.php). The experimental results can be obtained by running the source codes of the proposed method (please contact Huaping Guo (hpguo@xynu.edu.cn or hpguo_cm@163.com) to obtain the source code).
